# An international framework for clinical translation of molecular classifiers in osteosarcoma

**DOI:** 10.1038/s41698-026-01456-4

**Published:** 2026-05-05

**Authors:** Amanda E. Marinoff, Michaela Nathrath, David S. Shulman, Sarah B. Whittle, Jovana Pavisic, Dimitrios Spentzos, Catherine M. Albert, Joshua O. Nash, Adam Shlien, Isidro Cortés-Ciriano, Antonin Marchais, Eunice López-Fuentes, Christina Curtis, E. Alejandro Sweet-Cordero, Richard Gorlick, Natalie DelRocco, Rashmi Chugh, William D. Tap, Adrienne M. Flanagan, Nathalie Gaspar, Brian D. Crompton, Damon R. Reed, Katherine A. Janeway, Patrick J. Grohar, J. Andrew Livingston, Ryan D. Roberts

**Affiliations:** 1 Division of Pediatric Oncology, Department of Pediatrics, University of California San Francisco, San Francisco, CA USA; 2 Department of Pediatrics and Children’s Cancer Research Center, Klinikum rechts der Isar, Technical University of Munich, School of Medicine, Munich, Germany, Klinikum Kassel, Department of Pediatric Oncology, Kassel, Germany; 3 Dana-Farber/Boston Children’s Cancer and Blood Disorders Center, Harvard Medical School, Boston, MA USA; 4 Texas Children’s Cancer and Hematology Centers, Department of Pediatrics, Baylor College of Medicine, Houston, TX USA; 5 Department of Pediatrics, Memorial Sloan Kettering Cancer Center, New York, NY USA; 6 Department of Orthopedic Surgery, Division of Orthopedic Oncology, Massachusetts General Hospital Cancer Center, Harvard Medical School, Boston, MA USA; 7 Division of Pediatric Hematology/Oncology, Department of Pediatrics, University of Washington, Seattle, WA USA; 8 Genetics and Genome Biology Program, The Hospital for Sick Children, Toronto, Canada; 9 European Molecular Biology Laboratory, European Bioinformatics Institute, Hinxton, UK; 10 Somatic Genomics Programme, Wellcome Sanger Institute, Hinxton, UK; 11 INSERM U1015, Gustave Roussy, Paris Saclay University, Villejuif, France; Department of Pediatric and Adolescent Oncology, Gustave Roussy, Villejuif, France; 12 Stanford Cancer Institute, Stanford University School of Medicine, Stanford, CA USA; 13 Division of Pediatrics, University of Texas MD Anderson Cancer Center, Houston, TX USA; 14 Department of Sarcoma Medical Oncology, The University of Texas MD Anderson Cancer Center, Houston, TX USA; 15 Department of Population and Public Health Science Medicine, Keck School of Medicine, University of Southern California, Los Angeles, CA USA; 16 Department of Internal Medicine, University of Michigan, Ann Arbor, MI USA; 17 Department of Medicine, Memorial Sloan Kettering Cancer Center, New York, NY USA; 18 Research Department of Pathology, Cancer Institute, University College London, London, UK; 19 The Royal National Orthopaedic Hospital, Stanmore, Middlesex UK; 20 Department of Oncology for Child and Adolescent, Gustave Roussy Cancer Campus, Villejuif, France; 21 Department of Pediatrics, Division of Pediatric Hematology Oncology, University of Michigan Medical School, CS Mott Children’s Hospital, Ann Arbor, MI USA; 22 Division of Hematology, Oncology, and BMT, Department of Pediatrics, Nationwide Children’s Hospital and The Ohio State University, Columbus, OH USA

**Keywords:** Biomarkers, Cancer, Computational biology and bioinformatics, Genetics, Oncology

## Abstract

Despite well-recognized biological heterogeneity, osteosarcoma has been treated as a single disease for over four decades with minimal improvement in survival. Clinical features are inadequate for risk stratification, and no molecular classifiers guide therapy. An international working group evaluated candidate prognostic biomarkers for clinical translation. Pre-treatment circulating tumor DNA is positioned for clinical implementation, while additional classifiers warrant prospective validation. This work establishes a path to risk-adapted, biologically informed treatment.

## Introduction

Osteosarcoma, the most common primary bone tumor in children, adolescents, and young adults, is a highly aggressive cancer with treatment strategies and outcomes that have remained largely unchanged for over four decades^1–6^. The current standard of care combines intensive multi-agent chemotherapy with local control surgery. Despite the well-recognized biological heterogeneity of osteosarcoma, this approach does not incorporate clinical or molecular features for risk stratification or therapeutic selection. Consequently, an estimated 10-20% of patients likely would survive with surgery alone yet receive intensive multi-agent chemotherapy with associated acute toxicity and long-term sequelae^7^. Conversely, 30-40% of patients with localized disease and 80% of patients with metastatic disease die of osteosarcoma, despite this intensive therapy^3,4,8^.

Metastatic status and histologic response to neoadjuvant chemotherapy remain the most established prognostic factors, but they do not fully reflect underlying tumor biology or enable effective risk-adapted treatment^4,5,9,10^. Advances in molecular technologies have expanded our understanding of osteosarcoma heterogeneity and increasingly support the characterization of biologically and clinically distinct subgroups. However, findings remain fragmented, limiting their incorporation into prospective trials. A coordinated framework is needed to prioritize and integrate molecular features for clinical risk stratification and support biology-informed treatment approaches in osteosarcoma.

Molecularly-based stratification has facilitated risk-adapted strategies and improved survival in other malignancies, including breast cancer^11^, acute leukemias^12,13^, neuroblastoma^14^, and rhabdomyosarcoma^15^. Recent successful international efforts in Ewing sarcoma provide a framework for evaluating candidate molecular classifiers in rare pediatric, adolescent and young adult (AYA) cancers^9,10^. Building on these precedents, we established an international working group to assess molecular features for risk stratification in newly diagnosed osteosarcoma in children and AYA. In this review, osteosarcoma refers to conventional high-grade bone tumors defined by malignant osteoid production^16^.

## Clinical prognostic features in osteosarcoma

The presence or absence of metastatic disease at diagnosis remains the strongest clinical predictor of survival in osteosarcoma. Patients with localized disease have a 5-year overall survival (OS) of approximately 65–70%^4,8^. Those with metastatic disease at diagnosis face significantly worse outcomes, with a 5-year OS of approximately 20%^1,2,4,5,8,17^. Metastases are present in 15–20% of patients at diagnosis and most commonly involve the lungs or distant skeletal sites. Within the metastatic group, outcomes are heterogeneous, with 5-year event-free survival (EFS) ranging from 11–46%, reflecting variation in metastatic burden, site, and resectability^2,5,18–20^.

Among patients with metastatic disease, those with isolated pulmonary metastases amenable to complete surgical resection have the most favorable outcomes^2^. Conversely, patients with unresectable skeletal metastases, particularly those with multifocal lesions, face especially poor outcomes, with greater than 85% succumbing to disease within two years^21,22^. The prognostic significance of indeterminate pulmonary nodules remains uncertain, and there is no internationally accepted definition of pulmonary metastatic disease at diagnosis^23^. Skip metastases, defined as synchronous tumor foci anatomically distinct from the primary lesion, occur in 1–8% of cases and are associated with outcomes intermediate between localized and metastatic disease, with more favorable prognosis among patients with intraosseous skip lesions^24–26^. Complete resection of both the primary tumor and metastatic lesions, when achievable, remains critical for long-term survival in all metastatic contexts^2,5,27^.

Tumors in the axial skeleton, particularly the pelvis, are associated with worse outcomes than extremity primary tumors^27–30^. This may reflect both challenges in achieving complete surgical resection and potential differences in tumor biology. Larger primary tumor size is also associated with poorer outcomes, though inconsistencies in defining and measuring tumor size have hindered its incorporation into risk stratification^3,4,31^. Elevated pre-treatment serum lactate dehydrogenase and alkaline phosphatase are also linked to worse survival in some studies, though their independent prognostic value is unclear when accounting for disease stage and tumor size^32,33^. Other clinical features, including patient sex, age, histologic subtype, and pathologic fracture, have demonstrated inconsistent associations with outcome and generally confer limited independent prognostic value^2–5^.

Histologic response to neoadjuvant chemotherapy is a well-established prognostic factor, with ≥90% tumor necrosis associated with improved OS and EFS^1,2,4,8,34,35^. However, approximately 20% of patients with good histologic response die of osteosarcoma, while up to half of patients with poor response are long-term survivors^1,8,34,36^. Moreover, histologic response can only be assessed after more than a third of planned chemotherapy is delivered, and efforts to intensify therapy for poor histologic responders have not improved survival^1,37^.

The ongoing Children’s Oncology Group (COG) phase II/III trial AOST2032 (NCT05691478) defines high-risk patients with newly diagnosed osteosarcoma as those with metastases, an unresectable primary tumor or a pelvic primary tumor. In this trial, patients are stratified into risk cohorts for analysis, and both groups are eligible for the same therapeutic randomization. Although these clinical features are reproducibly associated with outcome, they do not capture the biological drivers of response and resistance or reliably distinguish risk groups. Effective risk-adapted therapy will require integration of these clinical factors with emerging molecular features associated with outcome.

## Evaluation strategy for molecular risk classifiers

**Fig. 1:**
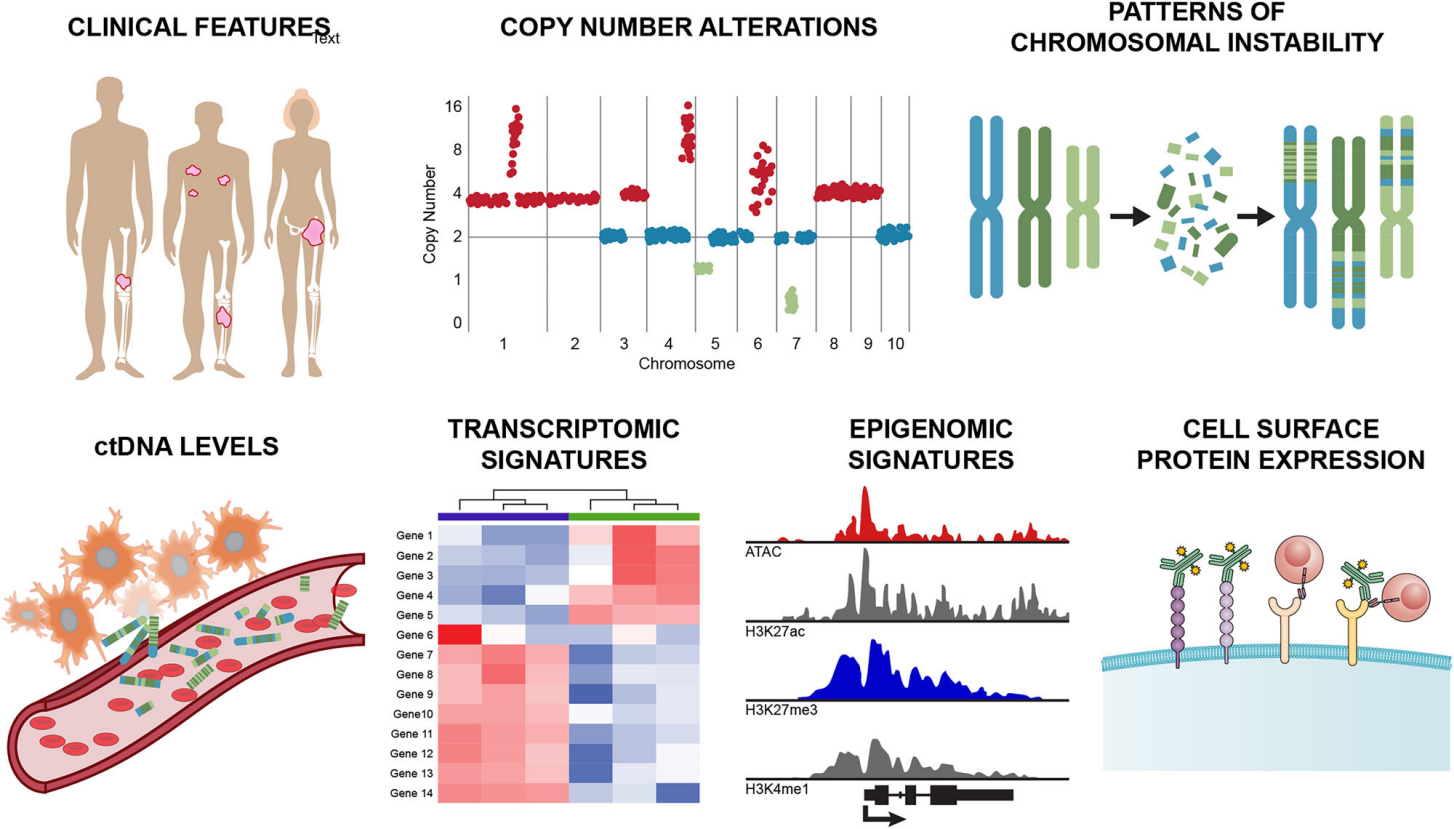
Schematic overview of candidate molecular classifiers for risk stratification in osteosarcoma. The working group evaluated clinical features and molecular features across five domains: circulating analytes (ctDNA), genomics (including copy number alterations and patterns of chromosomal instability), transcriptomics, epigenetics, and cell surface proteins.

We established an international working group of osteosarcoma experts to evaluate candidate molecular classifiers for risk stratification at diagnosis. Classifiers were identified based on published associations with clinical outcomes and organized into five domains: genomics, transcriptomics, epigenetics, cell-surface proteins, and circulating analytes (Fig. 1).

For each classifier, we considered the strength and statistical significance of associations with OS and/or EFS, independent prognostic value, reproducibility, generalizability, and clinical feasibility. Table 1 summarizes key supporting studies for each classifier, with expanded details in Supplemental Table 1. Data presented reflect those reported in the original studies.

**Table 1:** Summary of molecular classifiers for risk stratification in osteosarcoma with evidence level assignments and key supporting studies

Molecular feature	Study & Cohort(s) (n)	Method, high-risk group definition (frequency)	Key Reported Outcomes	Evidence level
**CIRCULATING TUMOR ANALYTES**				
**Pre-treatment ctDNA**	Audinot 2024 OS-2006 (178)	ULP-WGS, diagCPA score >0.6 (41%)	PFS: HR 3.5 (95% CI 1.60-7.5), *p* = 0.002; OS: HR 3.51 (95% CI 1.32-9.3), *p* = 0.012^a^	4
	Shulman 2018 AOST06B1^b^	ULP WGS, Detectable ctDNA (57%)	Each 1% increase in ctDNA: OS: HR 1.09 (95% CI 1.06-1.14), *p* < 0.001^b^	
**GENOMIC CLASSIFIERS**				
**MYC (8q24)**	Nagy 2025 DFCI/GAIN (105^d^)	16% amp, 22% high exp, 8% both	Amp: 3y-OS 27% vs 74%, HR 3.4, *p* < 0.0001; high exp: 3y-OS 35% vs 77%, HR 4.9, *p* < 0.0001; amp + high exp vs non-amp + low exp: 3y-OS 0% vs 79%, HR 17.7, *p* < 0.0001^a^	3
	van Ewijk 2025 Princess Máxima (48)	RNA-seq, high exp >57.31 cpm (31%); WES, amp: CN > 7	High exp: EFS: HR 3.38 (95% CI 1.71-6.66), OS: HR 2.88 (95% CI 1.22-6.76)^a^; amp not associated with OS or EFS	
	Marinoff 2023, **DFCI/GAIN (92);** *MSKCC*^b^ *(86)*	Targeted NGS, **CN** > **7 (12%);** CN > 2-fold change (12%)	**3y-OS: 34% vs 70%, p** = **0.034**; *3y-OS 56% vs 85%, p* = *0.046*^b^	
	De Noon 2021, TARGET (80) + ICGC (54)	SNP array, CN/ploidy ratio >2 and size <2 Mb (6%)	Amp: OS significantly worse, *p* < 0.001	
	Jiang 2022 Shanghai Gen Hospital (121)	Multi-omics; iCluster4 =MYC-driven (17%)	5y-OS: ~40% (worst of four subgroups), *p* = 0.03^a^	
	Smida 2010 German CCG (45)^**c**^	SNP array, AMP > 2-fold (15.6%);	OS: *p* = 0.01; EFS: *p* = 0.003	
**CDK4** **(12q14)**^**c**^	Zhou 2018 MGH + DFCI/Harvard (54)	IHC, staining score ≥3 (NR)	OS: ~40% vs 80%, *p* = 0.01	2
**Genome-wide LOH**^**c**^	Valle-Inclan 2025 Multi-cohort^f^ (248)	WGS, High LOH; LOH > 30% (NR)	PFS: HR 54.2 (95% CI 7.5-391), *p* = 0.002^a^	2
**TRANSCRIPTOMIC CLASSIFIERS**				
**G1/G2 signature**	van Ewijk 2025 Princess Máxima (48)	RNA-seq, G2 per Marchais et. al, 2022 (50%)	EFS: HR 3.32 (95% CI 1.34-8.21); OS: HR 4.07 (95% CI 1.19-13.9)^a^	3
	Marchais 2022, **OS2006 (79);** *TARGET (82), OS2006 (96*^*e*^*);*	RNA-seq, G2: above median 15-gene signature score (55.6%, *65%*) NanoString	**3y-OS: 100% (G1) vs 67.8% (G2), HR 6.33 (95% CI 1.66-24.1), p** = **0.00042**^a^; *confirmed in TARGET (p* = *0.00039) and holdout OS2006 (p* = *0.023)*	
**OTTER**	Comitani 2023 Multi-cohort from the Treehouse Childhood Cancer Initiative (107)^g^	RNA-seq (unsupervised RACCOON clustering algorithm)	4 clusters with differential survival; *p* = 5.56 ×10-5	3
**TME signature**	Palmerini 2025, **ISG/OS-2**^b^ **(62),** *TARGET*^b^ *(62), GSE33382*^b^ *(57)*	**NanoString PanCancer Immune panel,** *RNA-seq, Microarray*	**5y-OS: 47% vs 92%, HR 10.6 (95% CI 3.77-29.73) p** = **3×10⁻⁶**^a^^,b^; *TARGET-OS: OS: HR 8.68 (95% CI 1.85-40.59); GSE3382: OS: HR 18.63 (95% CI 1.88-193.70)*,	3
**EPIGENETIC CLASSIFIERS**				
**DNA methylation profile**	Lietz 2022 **TARGET (83),** *AECM (15)*	**HumanMethylation450K,** *HELP-tagging*; Hypermethylated cluster by median split (50%) for both cohorts	**Non-metastatic median RFS: 104.7mo vs 63.5mo; metastatic: 26.7mo vs 2.3mo; pooled p** = **0.006**^a^**;** *highly hypermethylated: 5-yr EFS: OR 9.3 (p* = *0.119)*	2
	Rosenblum 2015, AECM (15)	HELP-tagging, Hypermethylated: cutoff angle 50 (67%)	100% of patients with global hypermethylation relapsed; High TLR4 promoter methylation: 5y-EFS *p* = 1.7×10⁻⁶	
**CELL SURFACE PROTEINS**				
**CXCR4 expression**	Kusuma 2022, Meta-analysis (940)	IHC; scores range ≥2 to ≥5	OS: pooled HR 2.13 (95% CI 1.78-2.55), *p* < 0.001; metastasis: OR 4.01 (95% CI 1.58-10.18), *p* = 0.003	2
**B7H3**	Wang 2018 (37), Wang 2013 (61) Hebei Medical University	High sB7H3: > 60.94 ng/mL (92%); B7H3 protein: IHC score >3 (61%)	Tissue: median OS: 47.1mo vs 58.3mo, *p* = 0.005; serum: median OS 48.7mo vs 70.6mo^a^	2
**LRRC15**	Cui 2021 UCLA cohort (69)	IHC, score >2+ (74%)	5-yr OS: 72 vs. 45%, HR 2.6 (95% CI 1.4–4.3), *p* = 0.002^a^	2

**Fig. 2:**
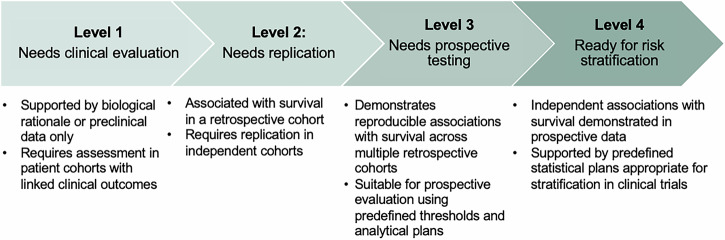
Evidence level framework for molecular classifiers. Classifiers were assigned to one of four levels based on strength of clinical association, reproducibility across cohorts, and clinical feasibility.

The cohorts and number of samples in each reflect only those included in survival analyses. In studies with multiple cohorts, **bold** text indicates discovery cohort, *italicized* text indicates the validation cohort. Additional studies and details on cohort characteristics, treatment, specimen types, and outcomes are provided in Table S1. Frequency refers to the proportion of cases in the high-risk group for a given study. Evidence level reflects consensus of the working group based on the criteria outlined in the methods and Fig. 2.

*AECM* Albert Einstein College of Medicine, *amp* amplification, *CCG* Clinical Cooperation Group, *CN* copy number, *COG* Children’s Oncology Group, *ctDNA* circulating tumor DNA, *DFCI* Dana–Farber Cancer Institute, *diagCPA* ctDNA quantification at diagnosis, *EFS* event-free survival, *exp* expression, *GAIN* Genomic Assessment Informs Novel therapy iCat2 study, *HR* hazard ratio, *IHC* immunohistochemistry, *LOH* loss of heterozygosity, *MGH* Massachusetts General Hospital, *NGS* next-generation sequencing, *NR* not reported, *OR* odds ratio, *OS* overall survival, *OS2006* OS2006 trial (NCT00470223), *PFS* progression-free survival, *RFS* relapse-free survival, *TARGET* Therapeutically Actionable Research to Generate Effective Treatments Osteosarcoma project, *TME* tumor microenvironment, *ULP-WGS* ultra-low-pass whole-genome sequencing, *WGS* whole-genome sequencing.

^a^Adjusted in multivariable model (MV) accounting for metastatic status (see Supplement for all variables included in MV models). HR and p-values reflect adjusted measures.

^b^Newly diagnosed, localized cohort.

^c^Smida et. al, 2010 also demonstrated the following associations: CDK4 amp [>2-fold change (11%)] showed a trend toward relapse (0.07); LOH at 12q14 (22%) was associated with worse EFS (*p* = 0.002); 6p21-12 AMP[ > 2-fold change (15.6%)was associated with worse EFS (*P* = 0.018); High total LOH score [SNPs with LOH > 1,500 (53%)] showed a trend toward worse OS (ns).

^d^70 unique samples, 35 overlapping with Marinoff et. al, 2023 cohort.

^e^Hold-out set not overlapping with OS2006 discovery cohort.

^f^The following cohorts were pooled in Valle Inclan et al., 2025: Behjati/PCAWG; G100K; MDACC; Kidsfirst; TARGET; UCL.

^g^Includes samples from TARGET(*n* = 59), ICGC (*n* = 5), and The Cancer Genome Atlas (*n* = 4).

Classifiers were assigned to one of four evidence levels (Fig. 2, Table 1). Level 1 includes biological classifiers supported by preclinical data that require assessment in patient cohorts with linked clinical outcomes. Level 2 includes classifiers associated with survival in retrospective studies that require replication in independent cohorts. Level 3 includes classifiers with reproducible associations with survival across multiple retrospective cohorts and that are suitable for prospective evaluation using predefined statistical plans. Level 4 includes classifiers with independent prognostic value demonstrated in at least one prospective cohort and that are ready to inform risk stratification in upcoming clinical trials.

Evidence levels reflect the strength and reproducibility of clinical associations rather than the specific assay platforms, analytic methods, or thresholds used to define risk groups in prior studies. Accordingly, a classifier may be appropriate for prospective evaluation even when evidence was generated using heterogeneous approaches, as formal risk group definitions will be established through multivariable modeling in prospective datasets. In line with convention in clinical literature, we refer to the relationship between clinical outcomes and the molecular classifier of interest without the influence of other confounding variables as independent prognostic value. To avoid terminology with regulatory implications, we refer to each candidate as a “molecular classifier” rather than “biomarker.” This framework focused on classifiers with near-term relevance for risk stratification in newly diagnosed osteosarcoma.

## Summary of evidence for molecular risk classifiers

### Circulating tumor DNA in osteosarcoma

Circulating tumor DNA (ctDNA) refers to short ( ~ 150 bp) tumor-derived DNA fragments that circulate in peripheral blood and can be detected using multiple different methodologies. ctDNA-based testing relies on differentiating tumor-associated DNA from non-tumor DNA. The characteristic genomic instability of osteosarcoma, which is defined by widespread structural rearrangements and copy number alterations, has limited the use of pre-defined, mutation-based assays. Instead, most ctDNA studies in osteosarcoma have used approaches that leverage tumor aneuploidy, such **as** ultra-low-pass whole genome sequencing (ULP-WGS), to detect and quantify ctDNA^38–40^. Other methods for detection of ctDNA in osteosarcoma are emerging, including detection of cancer-specific methylation patterns and the use of cell-free DNA fragment size and patterns (fragmentomics)^41^.

Multiple studies have demonstrated the independent prognostic value of pre-treatment ctDNA burden in osteosarcoma using these approaches^38,42,43^. In patients treated on the French OS2006 study, pre-treatment ctDNA levels were significantly associated with survival in patients independent of metastatic status (HR = 3.5, *p* = 0.002 and 3.51, *p* = 0.012, for PFS and OS, respectively)^42^. The LEOPARD Study (Liquid Biopsy in Ewing sarcoma and osteosarcoma as a prognostic and response diagnostic) was developed to prospectively validate these early findings using ULP-WGS. The initial results of the LEOPARD study were presented at the ASCO Annual Meeting in 2024 and confirmed pre-treatment ctDNA burden as a prognostic biomarker among patients with localized osteosarcoma treated with MAP chemotherapy^43^. Reproducible associations with outcome across retrospective cohorts, together with emerging prospective confirmation, position ctDNA to inform risk stratification in prospective clinical trials in patients with newly diagnosed osteosarcoma.

### Focal genomic alterations

Focal alterations in oncogenes and tumor suppressors are a recurrent feature of osteosarcoma^44–50^, with several loci demonstrating reproducible associations with survival. These genomic features are attractive risk stratification candidates as they can be assayed using clinical platforms, though translation has been hampered by heterogeneity in analytic methods, specimen quality, and risk group definitions.

Amplification of *MYC* at the 8q24 locus demonstrates the most consistent independent association with poor prognosis across multiple retrospective cohorts using diverse platforms, including whole genome sequencing (WGS), single nucleotide polymorphism (SNP) arrays, and targeted next-generation sequencing (NGS) panels^47,49,51–53^. In a multi-institutional cohort enriched for patients with advanced osteosarcoma, *MYC* amplification was associated with significantly worse 3-year OS (34% vs 70%, *p* = 0.034) and confirmed in an independent cohort of patients with localized disease (56% vs 85%, *p* = 0.046). *MYC* overexpression at the RNA level has additionally been linked to poor outcomes independent of copy number and metastatic status^54^. *MYC* amplification also correlates with protein expression by immunohistochemistry (IHC), with both independently and synergistically predicting poor survival^55^. Integrative multi-omics analysis further identified a *MYC*-driven osteosarcoma subtype with the poorest prognosis among four defined subgroups, with 5-year OS below 40%^56^. Collectively, these findings support prospective evaluation of *MYC* status as a prognostic classifier with a prespecified statistical plan. The ongoing COG study AOSTNV01-Q, which is testing *MYC* copy number using a clinically deployable assay in diagnostic specimens, may inform feasibility and standardization for frontline testing^57^.

Alterations in the p16-CDK4-RB pathway have also been linked to poor outcomes. Preliminary findings from AOSTNV01-Q suggest that *CDK4* amplification (12q14) is associated with significantly increased odds of an event, though confidence is limited by the low numbers of *CDK4*-amplified tumors without an event^57^. Smida et al. reported a trend toward worse survival among *CDK4*-amplified tumors, with broader 12q alterations significantly associated with poor EFS^43^. However, interpretation of these findings is limited, as only five *CDK4*-amplified cases were included in the latter study, all with metastatic disease^43^. *CDK4* overexpression at the RNA and protein levels has also been associated with chemotherapy resistance, poor survival, and metastatic disease^58,59^. *CDKN2A/B* deletion at 9p21 and loss of p16 protein have also been linked to poor outcomes^56,60–62^. RB1 loss (13q14) was associated with worse survival in studies from the 1990s and early 2000s, but this relationship has not been reliably reproduced in recent cohorts using contemporary sequencing methods^63^. Across all p16-CDK4-RB pathway alterations, independent prognostic value remains unclear, and replication in additional cohorts with multivariable analysis is needed. Notably, *RB1* loss is generally mutually exclusive with *CDK4* and *CDKN2A/B* alterations^64^, while *CDKN2A/B* loss frequently co-occurs with deletion of adjacent genes such as *MTAP*^65^. These findings suggest that pathway-level risk assessment, rather than evaluation of individual alterations, may be required to define optimal risk assessment strategies.

Additional alterations that demonstrate early promise as prognostic features include amplifications of 6p21, which encompasses *VEGFA*, *RUNX2*, and *CCND3*^47^. Supporting evidence from protein expression studies demonstrates that VEGF overexpression by IHC is independently associated with poor survival across multiple cohorts^66^. Amplifications of *CCNE1* (19q12)^67^ and *IGF1R* (15q26)^68^ are also linked to poor prognosis in single cohorts. These findings are biologically plausible but require replication.

### Genome-wide loss of heterozygosity and chromosomal instability archetypes

Beyond focal events, genome-wide patterns of instability have also emerged as potential prognostic features in osteosarcoma. Advances in genome-wide profiling, particularly through WGS, have begun to resolve the mechanisms underpinning this complexity^44–46,50^ and to identify structural features with prognostic value. Two complementary approaches have emerged: genome-wide loss of heterozygosity (LOH), which quantifies the overall burden of allelic imbalance across the genome, and broader chromosomal instability archetypes that capture distinct evolutionary trajectories.

In a large retrospective cohort, WGS identified a biologically distinct subgroup defined by high genome-wide LOH, which was independently associated with OS (HR 54.2; 95% CI: 7.5–391; *p* = 0.002)^50^. In this study, other clinical and individual genomic features, including whole-genome duplication, structural variant burden, and complex genomic rearrangements, were not associated with outcome. Smida et. al demonstrated a trend toward worse survival in osteosarcomas with high total LOH burden and significantly worse outcomes when high total LOH was combined with specific arm-level chromosomal alterations^47^. In parallel, emerging data using WGS have defined chromosomal instability archetypes characterized by distinct rearrangement processes and accompanied by distinct microenvironments. These patterns capture a different dimension of genomic complexity than LOH, and early results suggest more complex instability patterns are independently associated with inferior outcomes.

Both approaches require replication in independent cohorts, and several gaps to clinical translation remain. Because these features require genome-wide resolution, they cannot be reliably captured by targeted sequencing panels. Clinical implementation will require WGS or alternative assays with robust structural variant and LOH detection that are adapted for clinical use, along with standardized bioinformatic pipelines and thresholds to ensure reproducible classification.

### Transcriptomic classifiers

The extensive genomic complexity and paucity of recurrently mutated genes in osteosarcoma present challenges to stratification using DNA-based methods alone^44,46,48^. RNA-sequencing (RNA-seq) can capture gene expression programs that integrate genetic, epigenetic, and microenvironmental influences, providing an alternative classification strategy.

Since the public release of the Therapeutically Applicable Research to Generate Effective Treatments Osteosarcoma (TARGET OS) dataset in 2019, numerous bulk RNA-seq-based signatures have been proposed to stratify survival^69–80^. Single-cell RNA-seq data have further delineated specific cell states that contribute to bulk transcriptomic profiles^81–83^. While these studies support gene expression as a biologically relevant driver of outcome, most used supervised classification methods that risk overfitting to TARGET and lack sufficient external validation. In addition, limited availability of RNA-seq data linked to clinical outcomes has led many studies to use microarray datasets to replicate findings, which complicates interpretation and cross-cohort comparison^69–79^.

Three recent studies have demonstrated reproducible survival associations across multiple cohorts using unsupervised methods^84–86^. First, RNA-seq profiling from the OS2006 trial revealed two prognostically distinct groups (G1 and G2), with favorable-outcome tumors enriched for immune signatures and poor-outcome tumors characterized by angiogenic and metabolic programs (3-year OS 100% vs. 67.8%; *p* = 0.00042)^85^. The G1/G2 classifier was validated in multiple independent datasets^54,85^ and will be incorporated as a minimization factor in the prospective FOSTER-CabOs trial (EU CT No: 2023-505575-69-00). Second, unsupervised analysis of a multi-cohort dataset identified four osteosarcoma transcriptomic subtypes reflecting distinct mesenchymal lineage programs and significant differences in overall survival (*p* = 5.56 ×10^-5^)^84^. A convolutional neural network called Oncologic TranscripTome Expression Recognition (OTTER) was developed to assign new tumors to these subtypes and is currently being evaluated in additional retrospective and prospective cohorts and adapted for FFPE^84,87^. Third, gene expression analysis of diagnostic samples from the ISG/OS-2 trial identified a tumor microenvironment signature that stratified patients with localized disease into two risk groups (5-year OS 35.7% vs. 89%; *p* < 0.0001), with validation in two additional cohorts^86^. Tumors with favorable outcomes were enriched for CD8 + T-cell migration and cytokine-mediated signaling programs.

These three classifiers have each demonstrated reproducible survival associations across multiple independent cohorts and warrant prospective validation with predefined statistical plans. Several other transcriptomic signatures have demonstrated strong prognostic performance within their respective training and internal test sets but require replication in independent external cohorts^73,76,79^. Given the growing number of reported transcriptomic classifiers and their methodological variability, systematic comparative evaluation across uniformly assayed cohorts will be needed to establish which approaches are most reproducible and clinically feasible. Whole transcriptome sequencing (WTS) is preferred over targeted panels when feasible, as it enables unbiased discovery, facilitates direct comparison across signatures, and avoids constraints of predefined gene sets. Although most work to date has relied on fresh frozen tissue, recent improvements in RNA capture and library preparation now enable high-quality WTS from FFPE^88^. Clinical implementation will require validating classifier performance and defining risk groups using FFPE-derived data.

### Epigenetic classifiers

Epigenetic alterations, including DNA methylation and chromatin accessibility changes, play key roles in transcriptional regulation and are increasingly recognized as likely drivers of osteosarcoma progression, metastasis, and therapeutic resistance^89–93^. These epigenetic features may reflect dynamic tumor states not captured by genomic or transcriptomic profiling alone and could serve as complementary classifiers for risk stratification.

DNA methylation profiling is a robust diagnostic and prognostic tool across diverse cancers^94–97^. In osteosarcoma, global hypermethylation is associated with poor chemotherapy response and inferior recurrence-free survival in the TARGET cohort (RFS, non-metastatic: 104.7 vs 63.5 months; metastatic: 26.7 vs 2.3 months; pooled log-rank *p* = 0.006), with similar findings in a smaller independent dataset using a different assay^89,98^. Multi-omics integration of methylation, genomic, and transcriptomic data has further improved prognostic performance^56^. While multiple platforms have demonstrated feasibility^99–101^ and several new technologies are emerging, formal comparison of platform performance and consensus on standardized assays and thresholds will be important for clinical translation. The prognostic value of specific methylation profiles requires replication in at least one additional large, independent cohort to ensure reproducibility before prospective validation.

Chromatin accessibility profiling provides additional insight into regulatory dynamics by identifying regions of open chromatin available for transcription factor binding^102,103^. Recent studies using the assay for transposase-accessible chromatin (ATAC)- seq have revealed chromatin remodeling events associated with metastatic competence and delineated chromatin-defined osteosarcoma subtypes with distinct transcriptional circuitry and drug sensitivity^104^. While these findings are preclinical, they highlight the potential for epigenetic approaches to stratify patients by prognosis and subtype-specific vulnerabilities. Translation will require further investigation in diagnostic patient samples linked with clinical outcomes and the development of clinically feasible assays.

### Cell surface proteins

Cell surface proteins represent an emerging area of osteosarcoma biology with translational relevance. While interest in these markers has primarily been driven by their therapeutic potential^105^, several have demonstrated reproducible associations with survival and warrant consideration as prognostic classifiers.

CXCR4, a chemokine receptor that mediates CXCL12-directed cell migration and metastatic homing, has demonstrated reproducible association with survival across multiple retrospective studies^106–110^. A meta-analysis of 12 studies found that high CXCR4 protein or mRNA expression was associated with increased risk of metastasis and poor OS (pooled HR = 2.13, 95% CI 1.78–2.55; *p* < 0.001), though independent prognostic value has not been clearly demonstrated^110^. Further work is also needed to determine whether IHC, RNA expression, or combined scoring approaches provide the most reliable and clinically meaningful measure of CXCR4 activity, and whether related features such as VEGF expression offer complementary prognostic information^111^.

Several additional cell surface proteins have demonstrated prognostic associations in osteosarcoma but require replication in additional cohorts. B7-H3 (CD276), an immune checkpoint molecule, has been linked to poor survival when assessed by IHC or elevated serum levels of soluble B7-H3^112,113^. LRRC15, a leucine-rich repeat protein involved in tumor–stromal interactions, has also been associated with inferior survival, increased metastatic risk, and poor histologic response when measured by IHC^114,115^. Importantly, the dual role of these features as potential prognostic markers and therapeutic targets may offer unique opportunities to bridge risk stratification with novel treatment strategies.

## Discussion

Despite decades of research, osteosarcoma remains one of the few malignancies without classifiers to guide risk stratification or treatment selection. The emergence of reproducible molecular features capable of stratifying osteosarcoma into clinically and biologically distinct subgroups signals a turning point. This review represents the first international effort to prioritize molecular risk classifiers for osteosarcoma and establish a framework for their translation.

Among candidate classifiers, pre-treatment ctDNA burden is the most mature feature, with prospective data supporting its use to guide risk stratification in clinical trials. *MYC* status and three transcriptomic classifiers have each demonstrated reproducible independent prognostic associations across multiple cohorts and warrant prospective evaluation with predefined statistical plans. Additional features show promise and require replication and/or demonstration of independent prognostic value, including CDK4 pathway alterations, genome-wide LOH, DNA methylation profiles, and several cell surface proteins (Tables 1 and S1). These molecular classifiers should ultimately be integrated with clinical prognostic factors, including metastatic status, tumor location, and resectability. While this review was focused, many additional molecular classifiers have been proposed and may merit further evaluation^74,77,82,116–118^.

The evidence levels assigned to each classifier are intended as a practical framework to guide next steps rather than fixed or hierarchical categories. Levels 2 and 3 exist on a continuum of evidence maturity, and studies in retrospective cohorts can proceed in parallel with prospective evaluation when feasible. Prospective validation of Level 3 classifiers should include multivariable analysis to establish or confirm independent prognostic value.

**Fig. 3:**
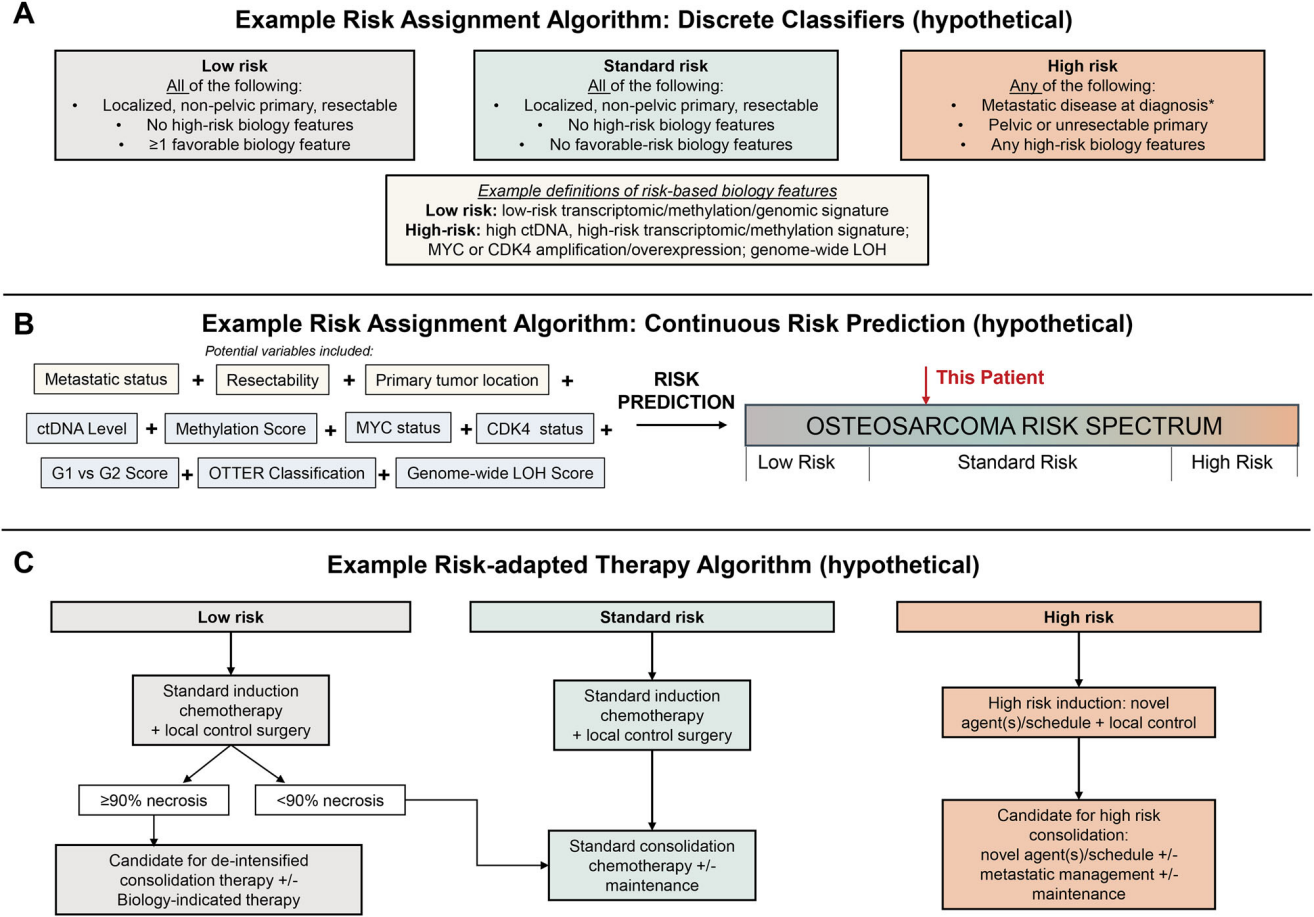
Hypothetical frameworks for implementing clinical and molecular risk classifiers in osteosarcoma. **A** Example rule-based algorithm using discrete risk categories. Features shown are illustrative. *Metastatic disease at diagnosis may not uniformly indicate high risk. **B** Example continuous risk prediction model integrating diverse clinical and molecular features. Features are shown additively for simplicity; relative contributions and final model structure remain to be established. **C** Hypothetical framework for future risk-adapted trials informed by prospectively validated classifiers and consensus treatment strategies.

**Fig. 4:**
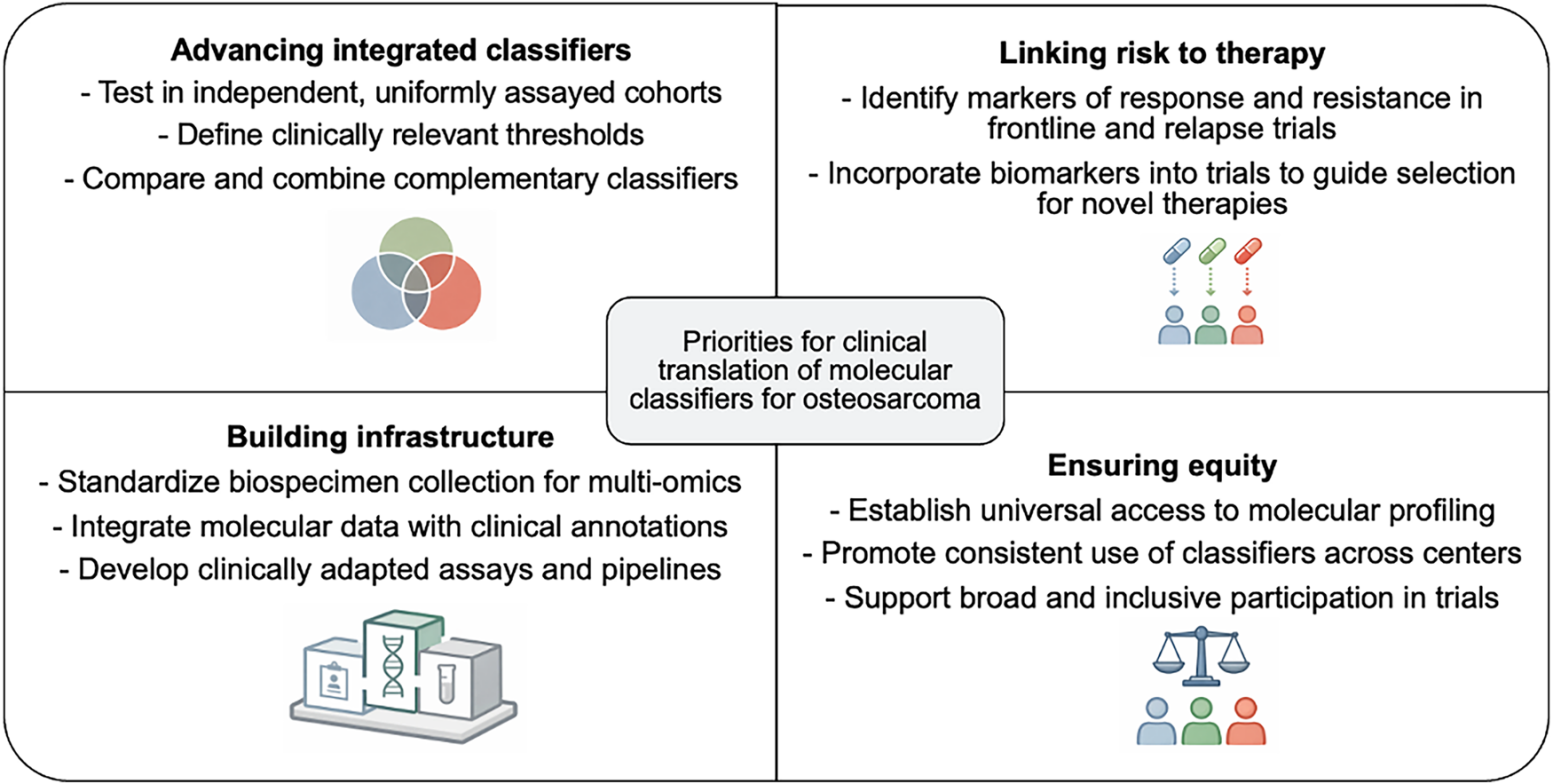
Priorities for clinical translation of molecular classifiers in osteosarcoma. The working group identified four interdependent priorities: advancing integrated classifiers, linking risk to therapy, building infrastructure, and ensuring equity.

To illustrate how these classifiers might eventually be applied, Fig. 3 presents two hypothetical approaches: a rule-based framework that groups patients by discrete classifiers (Fig. 3A) and a continuous approach that predicts risk on a probabilistic scale (Fig. 3B). These examples are not recommendations but rather conceptual tools to envision future possibilities. If validated, such frameworks could support trials investigating risk-adapted treatment strategies (Fig. 3C), such as earlier evaluation of novel therapies for high-risk patients and cautious treatment de-intensification for patients with favorable prognoses. Incorporation of such strategies into clinical trials would require prospective validation of risk groups and alignment on appropriate treatment approaches, in partnership with patient and family advocates. To accelerate progress, the working group identified four key priority areas (Fig. 4).

### Advancing integrated classifiers

Most classifiers to date have been developed in relatively small, heterogeneous cohorts using variable analytic methods and specimen types (Table S1) that preclude direct comparison and integration. It remains unclear whether different classification methods are selecting the same patient groups or whether each method captures distinct biological features that, when combined, could yield more precise stratification. Multi-omics assessment of individual features may further enhance prognostic performance compared to single-modality approaches^55,56^. While existing retrospective data can inform cross-study comparisons and hypothesis generation with appropriate consideration of patient- and specimen-level confounders, uniformly profiled cohorts will be essential to rigorously compare classifiers and establish optimal integration strategies for clinical implementation. When publishing integrated datasets, consistent annotation and reporting should include, where available, the timing of specimen collection (pre-treatment, post-neoadjuvant therapy, refractory disease, or post-therapy relapse) and key treatment exposures. These factors should be considered as potentially important co-variates when integrating retrospective cohorts. Systematic comparative analyses in uniformly profiled cohorts are needed to address these questions and determine optimal integration strategies.

### Linking risk to therapy

Although this review has emphasized molecular classifiers that stratify risk at diagnosis, their full impact will ultimately be realized by linking them to treatment strategies that improve survival. Achieving this goal will require deeper understanding of the biology underlying osteosarcoma subtypes to define therapeutically actionable vulnerabilities. Many prognostic features already suggest such opportunities: *MYC* and *CDK4* amplification are being explored as therapeutic targets^59,119–121^; immune-related transcriptional programs may identify subsets likely to benefit from immunomodulatory therapies^56,85,86,122–124^; B7-H3 and LRRC15 are under investigation as targets for antibody-drug conjugates and adoptive cellular therapies^125–127^; and chromosomal instability archetypes may reveal vulnerabilities to DNA damage response inhibitors^128^. Together, these examples highlight opportunities to align risk stratification with therapeutic development. Importantly, molecular features may inform risk stratification, therapeutic selection, or both, and optimal thresholds may differ by application. Prospective trials incorporating molecular features will need to account for these potential distinctions.

Linking prognostic classifiers to therapeutic strategies will require coordinated evaluation across trial settings. Because most new agents are first tested in relapse trials, correlative studies in this context provide critical opportunities to define markers of response and resistance, refine patient selection, and inform rational combination strategies. Over time, these efforts can facilitate incorporation of active agents into frontline regimens for high-risk patients. In parallel, molecular features linked to prognosis at relapse warrant investigation to inform trial design and guide prioritization of novel agents, particularly for the highest-risk subsets.

### Building infrastructure

The success of these efforts will require sustained investment in biospecimen collection, multi-omics profiling, and systematic integration of clinical and molecular data. Limited quantity and variable quality of historical banked specimens have hindered sequencing efforts. Recent consensus recommendations in bone sarcomas have outlined standards for biopsy planning, processing, and allocation to maximize clinical and research utility^129,130^. Building on these standards, future studies should prioritize pre-treatment diagnostic biopsies with comprehensive clinical annotations to enable classifier implementation at diagnosis, while specimens collected at relapse and progression are essential for understanding tumor evolution and resistance mechanisms. Applying broad profiling platforms such as WGS and WTS to detect emerging prognostic features^50,54,84,85^ may require optimizing clinical workflows for frozen tissue collection or adapting assays for FFPE.

Inconsistent and incomplete clinical annotation remain major barriers. Accurate interpretation of molecular classifier performance requires detailed knowledge of the clinical context in which a specimen was obtained, yet many studies lack comprehensive documentation of patient characteristics, disease features, treatment, and specimen metadata. Even when metastatic status is captured, it is typically reported in a binary manner, obscuring heterogeneity in metastatic burden, anatomic distribution, and resectability that influence outcomes. Interpretation of molecular features requires knowledge of specimen collection timing and prior treatment exposures, both of which are inconsistently reported. This is particularly relevant for genomic complexity-based features, which may reflect therapy-associated selection or genome remodeling in addition to intrinsic biology^131,132^. Survival measures are also defined inconsistently across studies (Table 1). Taken together, standardized collection of clinical covariates and outcome measures is a prerequisite to conducting comparative studies and developing robust classifiers. Coordinated international efforts to link clinical annotation to banked specimens and harmonize clinical data dictionaries represent foundational investments that will accelerate this progress^133,134^. Artificial intelligence-based methods offer increasing opportunities to integrate multi-omic and clinical data, enhance prediction models, and standardize annotation as datasets grow in scale and complexity. These approaches may also be valuable in settings of diagnostic uncertainty, such as indeterminate pulmonary findings or equivocal histopathology.

### Ensuring equity

National and international initiatives are beginning to provide infrastructure for standardized molecular profiling. The National Cancer Institute’s Molecular Characterization Initiative is prospectively generating multi-omics data across several pediatric cancers but does not currently include osteosarcoma. In parallel, the Fight Osteosarcoma Through European Research (FOSTER) consortium is harmonizing existing genomic and clinical datasets across countries. These efforts extend beyond discovery; they create the conditions for equity by ensuring broad access to molecular profiling, supporting consistent classifier application, and linking patients to cooperative research networks. Incorporating osteosarcoma into such initiatives is essential to enable prospective evaluation of classifiers in parallel and ensure that advances translate into benefit for all patients, regardless of treatment setting, demographic background, or geography.

## Conclusion

Osteosarcoma is poised to move beyond treatment strategies that do not reflect the profound clinical and biological heterogeneity of the disease. Realizing this transition will require coordinated investment in universal molecular profiling, clinical-genomic integration, and trials designed to incorporate clinically and molecularly defined subgroups. These initiatives must advance in parallel with efforts to develop more effective therapies, particularly for patients with high-risk disease. Importantly, once molecular classifiers are validated for risk stratification, prospective studies will need to demonstrate that their implementation not only predicts clinical outcomes but meaningfully improves them. Together, these efforts herald a long-overdue shift toward biologically informed treatment strategies in patients with osteosarcoma.

## Supplementary information

Supplementary Information

## Data Availability

No new data were generated during this study. All data discussed in this manuscript were obtained from previously published studies and are available from the original sources cited.
