# Supplementary material for: An international framework for clinical translation of molecular classifiers in osteosarcoma
Source: NPJ Precis Oncol. 2026 May 5;10:266. doi: 10.1038/s41698-026-01456-4 (PMC13346500; doi:10.1038/s41698-026-01456-4)
Supplement: Supplementary file 1 — Supplementary Information [file 41698_2026_1456_MOESM1_ESM.pdf]

Supplemental Table 1. Detailed characteristics of molecular classifiers for risk stratification in osteosarcoma.

Molecular Feature	Study & Cohort(s) (n)	Population Disease Setting | Age | Treatment	Sample Type	Assay	Definition Of High-Risk Group (Freq)	Key Reported Association with Survival	Evidence for Independent Prognostic Value
**Pre-treatment ctDNA**	Audinot 2024 OS-2006 (178)	Newly diagnosed, localized + metastatic| Age: 14.7 (4.7-50.4) | MTX + IE or Doxorubicin, cisplatin, ifosfamide +/- zoledronate	Pre-treatment plasma	ULP-WGS	diagCPA score >0.6 (41%)	Pre-treatment ctDNA independently associated with worse PFS: HR=3.5 (1.60-7.5), p=0.002; OS: HR=3.51 (1.32-9.3), p=0.012	**Yes**. MVA adjusted for metastatic status, age, sex, and histologic response.
**Pre-treatment ctDNA**	Shulman 2024* LEOPARD (133)	Newly diagnosed, localized | 14.2 (4.2-30.3) | MAP	Pre-treatment plasma	ULP-WGS	Analysis ongoing with results forthcoming	Analysis ongoing with results forthcoming	**Partial**. Study limited to localized, non-pelvic disease. Full methods forthcoming.
**Pre-treatment ctDNA**	Shulman 2018 AOST06B1 (72)	Newly diagnosed, localized |13.6 (5.6 22.4) | MAP +/- other	Pre-treatment plasma	ULP-WGS	ctDNA as a continuous variable (57% with detectable ctDNA)	Among 41 patients with detectable ctDNA, HRs for each unit increase in percent ctDNA: 1.06 ($p < 0.001$) and 1.09 ($p < 0.001$) for EFS and OS, respectively	**Partial.** Study limited to localized, non-pelvic disease.
**MYC amplification (8q24)**	Marinoff 2023, **PROFILE/ GAIN (92);** *MSKCC (86)*	**Newly diagnosed + recurrent, localized + metastatic | 13 (6-28)** | MAP; Newly diagnosed, localized |	Diagnostic biopsies, primary tumor resection	Targeted NGS (**OncoPanel,** MSK-IMPACT)	**MYC CN >7 (12%);** MYC >2-fold change (12%)	3y-OS: **34% vs 70% (p=0.034)**; 56% vs 85% (p=0.046) in validation	**Partial**. Association held in localized disease subgroups in primary (n=55) and validation cohorts (n=86). No MVA performed.
**MYC amplification (8q24)**	De Noon 2021, TARGET (80) + ICGC (54) (pooled)	Newly diagnosed, localized + metastatic	Diagnostic biopsies, relapsed specimens, primary tumor resections	SNP array (Affymetrix 6.0)	CN/ploidy ratio >2 and size <2Mb (ICGC: 15%, TARGET: 1%, total: 6%)	MYC amp associated with significantly worse OS (p<0.001)	**Partial.** Stratified by metastatic status with pooled p-value reported. No MVA performed.
**MYC amplification (8q24)**	Smida 2010, German CCG (45)	Newly diagnosed, localized + metastatic| 14 (4-51) | MAP +/- other	Diagnostic biopsies	SNP array (Affymetrix 10K2)	8q24(MYC) amp >2-fold (15.6%)	8q24 amp associated with significantly worse EFS (p=0.0004). 7/7 cases with 8q24 amp relapsed within 2 years.	**Partial.** Association held in localized disease subgroup only.
**MYC protein expression**	Nagy 2025 DFCI/ GAIN (105#)	Newly diagnosed + recurrent, localized + metastatic | 14 (IQR 11-17) | MAP	Diagnostic biopsies, primary tumor resection	Targeted NG (OncoPanel) and IHC	Amp: MYC CN > 7; exp >175 H-score (16% amp, 22% high exp, 8% both)	Amp: 3y-OS 27% vs 74%, HR 3.4, p<0.0001; high exp: 3y-OS 35% vs 77%, HR 4.9, p<0.0001; amp + high exp vs non-amp + low exp:	**Yes**. MVA performed. Associations held in both localized and metastatic disease subgroups in stratified analysis with stratified p-values reported.

						3y-OS 0% vs 79%, HR 17.7, p<0.0001	
**MYC RNA expression**	van Ewijk 2025, Princess Máxima (48)	Newly diagnosed, localized + metastatic Age: 14 (1-17)| MAP	Diagnostic biopsies, primary tumor resection	RNA-seq	MYC expression >57.31cpm (31%)	MYC expression independently associated with worse EFS: HR=3.38 (1.71-6.66); OS: HR=2.88 (1.22-6.76)	**Yes.** MVA adjusted for metastatic status, MYC amplification, histologic response, and G1/G2 signature.
**MYC RNA expression**	Jiang 2022, Shanghai General Hospital (121)	Newly diagnosed, localized + metastatic |Age: 52% <18 (6-67) | MAP	Primary tumor resection specimens	Multi-omics (WES, RNA-seq, methylation)	iCluster4 (MYC-driven) by integrative clustering (17%)	iCluster4 (MYC-driven) associated with worst 5y-OS (~40%) of four subgroups (p=0.03)	**Partial.** Association held within Enneking Stage IIB subgroup. No MVA performed.
**CDK4 (12q14)**	Whittle 2025*, AOSTNV01-Q (65)	Newly diagnosed Age: NR MAP +/- other	Diagnostic biopsies	SNP microarray (OncoScan)	Ratio of CDK4 to chr. 12 copies (NR)	Ongoing analysis, results forthcoming	**Partial**. Case-control design. Full methods forthcoming.
**CDK4 (12q14)**	Zhou 2018, MGH + DFCI/. Harvard tissue bank (54)	Newly diagnosed + recurrent, localized + metastatic Age: 24.5 (6-77) MAP	Primary, recurrent, metastatic specimens	IHC	High CDK4 expression: staining score ≥3 (NR)	Elevated CDK4 expression associated with worse OS (~40% vs 80%, p=0.01)	**No.** No analysis performed to assess independent prognostic value.
**CDK4 (12q14)**	Smida 2010, German CCG (45)	Newly diagnosed, localized + metastatic| 14 (4-51) | MAP +/- other	Diagnostic biopsies	SNP array (Affymetrix 10K2)	12q14 (CDK4) amp >2-fold (5/11); LOH at 12q14 (10/22%)	5/5 cases with CDK4 associated with lung metastases (p=0.01) and relapse(p=0.07); LOH at 12q14 associated with poor EFS (p=0.002)	**No.** Stratified analysis not possible; all cases with 12q14 amplification had metastatic disease. No MVA performed.
**6p12-21 (VEGFA, RUNX2, CCND3)**	Xin 2022 Meta-analysis of 25 studies (1278)	Newly diagnosed + recurrent, localized + metastatic | Weighted median 18.5 (5-69) | NR	Primary, recurrent, metastatic specimens	VEGFA expression predominantl y by IHC (1 study RT-PCR, 2 studies ELISA)	10-50% stained cells positive for VEGF	Elevated VEGFA expression associated with poor OS (pooled HR = 2.58; 95% CI, 2.09-3.19; P < 0.0001) and DFS (HR = 2.54; 95% CI, 1.84-3.50; p < 0.0001)	**No**. Few component studies included analyses to assess independent prognostic value.
**6p12-21 (VEGFA, RUNX2, CCND3)**	Yang 2011 Tianjin Medical University Cancer Institute and Hospital (50)	Newly diagnosed, localized + metastatic| <15-40y| Rosen T10 regimen	Primary tumors	VEGFA amplification by FISH; protein expression by IHC	VEGFA amplificatio n orange: green ratio >1 with >2 signals per nucleus (focal) or ratio =1 with >2 signals (large fragment) (64%); high exp: IHC score >2	VEGFA amplification and protein expression associated with poor DFS (p=0.042)	**No.** No analysis performed to assess independent prognostic value.
**6p12-21 (VEGFA, RUNX2, CCND3)**	Smida 2010 German CCG (45)	Newly diagnosed, localized + metastatic| 14 (4-	Diagnostic biopsies	SNP array (Affymetrix 10K2)	Amplificatio n: >2-fold change (22%)	Amplification was significantly associated with poor EFS (P = 0.018)	**Partial.** Association held in localized disease subgroup. No MVA performed.

		51) | MAP +/- other					
**RB1 (13q14)**	Ren 2015 Meta-analysis of 12 studies (491)	Newly diagnosed, localized + metastatic | Weighted median age 17.3 (11.6-24.5) | MAP +/- other	Diagnostic biopsies, primary tumor resection specimens	LOH (PCR, Southern blot), IHC	RB1 alteration (LOH or loss of pRB expression) (12-80% across studies)	RB1 loss associated with worse survival: RR=1.62 (95% CI 1.23-2.13), p=0.0006; increased metastasis: OR=3.95 (95% CI 1.86-8.38), p=0.0004; poor histologic response: OR=0.35 (95% CI 0.13-0.94), p=0.038	**No**. Few component studies included analyses to assess independent prognostic value.
**CDKN2A/p16 (9p21)**	Nielsen 1998 Massachusetts General Hospital (21)	Newly diagnosed, localized + metastatic | 26 (9-77) | NR	Diagnostic biopsies, primary tumor resection specimens	Comparative multiplex PCR for homozygous deletion; IHC for p16 and pRb	Homozygous CDKN2A deletion or loss of p16 expression (26% of high-grade)	5/19 (26%) high-grade osteosarcomas had CDKN2A/p16 alterations; mutually exclusive with RB/pRb alterations (p16-pRb pathway deregulated in 74% of high-grade tumors)	**No**. No analysis performed to assess independent prognostic value.
**Genome-wide LOH**	Valle-Inclán 2025 Behjati/PCAWG G100K MDACC Kidsfirst TARGET UCL (248)	Newly diagnosed + recurrent, localized + metastatic| 30 (4-89) | MAP +/- other (relapse)	Diagnostic biopsies, primary tumor resections, metastatic lesions and relapse	WGS	High LOH: >30% (NR)	PFS: HR 54.2 (95% CI 7.5-391), p=0.002; OS stratified by metastatic status, pooled p= 0.0008	**Yes.** MVA for PFS adjusted for metastatic status; sex, age, WGD, CGR, SNV, SV burden NS on univariate analysis. OS analysis stratified by metastatic status, pooled p-value reported.
**Genome-wide LOH**	Smida 2010, German CCG Osteosarcoma (45)	Newly diagnosed + recurrent, localized + metastatic Age: 14 (4-51)| NR	Diagnostic biopsies	SNP array (Affymetrix 10K2)	High LOH score: SNPs with LOH >1,500 (53%)	High LOH score: trend toward worse OS (ns); significantly associated with poor histologic response	**Partial.** Association held in localized disease subgroup. No MVA performed.
**G1/G2 transcriptomic signature**	Marchais 2022, **OS2006 (n=79)**; TARGET-OS (n=82), OS2006 (96[†]); exploratory: MAPPYACTS (32)	**Newly diagnosed, localized + metastatic | 15.4 (4.7-36.8) | MAP or MTX (n ¼ 69) or API-AI (n ¼ 10);** MAPPYACTS: relapsed.	Diagnostic biopsies (OS2006+ TARGET); Primary and metastatic at unspecified time points (MAPPYACTS)	RNA-seq, NanoString	**G2 by elastic net logistic regression model (56%); Higher proportion of G2 tumors in relapsed cohort (66%)**	**G2 associated with significantly worse OS and PFS; 3y-OS: 100% (G1) vs 67.8% (G2), p=0.00042; HR 6.327 (95% CI 1.657, 24.13).** Associations with OS and PFS confirmed in validation cohorts	**Yes.** MVA adjusted for metastatic status and histologic response; tumor size, treatment, pubertal status NS on univariate analysis.
**TME transcriptomic signature**	Palmerini 2025, **ISG/OS-2 (n=62)**, *TARGET (62), GSE33382 (57)*	**Newly diagnosed, localized | 14 (5-40) | MAP +/- mifamurtide**	Diagnostic biopsies	**NanoString PanCancer Immune panel,** *RNA-seq Microarray*	Hi-R: Risk score >2.419 using 21-gene signature (37%)	Hi-R significantly worse OS and EFS in discovery and validation cohorts; **5y-OS: 47% (Hi-R) vs 92% (Lo-R), $p=3\times10^{-6}$**	**Yes.** MVA adjusted for age, sex, histologic response, alk phos. Study limited to localized disease.

**OTTER**	Comitani 2023 Multi-cohort from the Treehouse Childhood Cancer Initiative (107)^	Newly diagnosed + recurrent, localized + metastatic	Diagnostic biopsies, primary tumor resections	RNA-seq	Unsupervised hierarchical clustering	4 clusters with differential survival; p = 5.56 x 10-5	**No.** No published analysis performed to assess independent prognostic value. Ongoing analyses to address this.
**DNA methylation subtype**	Lietz 2022, **TARGET (n=83)** AECM (n=15)	**Newly diagnosed, localized + metastatic Age: 14 (3-80) | MAP**	Biopsies |Naïve|	HumanMethylation450K, HELP-tagging	Hypermethylated cluster (by unsupervised hierarchical clustering) (50%)	Median RFS (non-metastatic): 104.7mo vs 63.5mo (hypermethylated); metastatic: 26.7mo vs 2.3mo; pooled p=0.006	**Partial**. Stratified analysis by metastatic status; pooled p-values reported. Individual CpG sites tested in separate MVA models adjusting for either chemoresponse or miRNA expression, but not metastatic status or other clinical factors simultaneously.
**DNA methylation subtype**	Rosenblum 2015, AECM from COG trials (n=15)	Newly diagnosed, localized + metastatic | 14 (11-17) | INT0133 (n=1), P9754 (n=9 patients) and AOST0121 (n=5).	Pretreatment biopsies (frozen)	HELP-tagging assay	Hypermethylation: cutoff angle 50 (67%)	Global hypermethylation associated with relapse; TLR4 promoter methylation significantly associated with 5y-EFS ($p=1.7\times10^{-6}$)	**Partial**. PCA with linear modeling showed methylation more strongly associated with relapse than metastatic status. No analyses performed to assess independent prognostic value.
**CXCR4 expression**	Kusuma 2022, Meta-analysis of 12 studies of single institution cohorts (940)	Newly diagnosed, localized + metastatic | Weighted median 18 (4-88) |MAP +/- other	Biopsies, resection| Pre-treatment	IHC (RT-PCR used in one study- REF Lavadere)	CXCR4 IHC scores variable (range ≥2 to ≥5)	CXCR4 expression associated with poor OS: pooled HR=2.13 (1.78-2.55), p<0.001; and metastasis: OR=4.01 (1.58-10.18), p=0.003	**No.** Few component studies included analyses to assess independent prognostic value.
**B7H3**	Wang 2013 Hebei Medical University (61)	Newly diagnosed, localized + metastatic| Age≤20 (n=34), >20(27) | NR	Primary tumor |Naïve| (FFPE)		B7H3 IHC score >3	High B7H3 protein expression was associated with significantly worse OS: median 47.1mo vs. 58.3mo (p=0.005).	**No.** B7H3 expression correlated with metastatic status. No analysis performed to assess independent value.
**B7H3**	Wang 2018 Hebei Medical University and Bethune International Peace Hospital (37)	Newly diagnosed, localized + metastatic| age≤10 (n=16), >10 (n=21) | NR	Pretreatment serum	ELISA	High sB7H3: > 60.94 ng/mL (92%); B7H3 protein: IHC score >3 (61%)	Median survival time for the OS patients with high- and low-sB7-H3 levels were 48.69 and 70.62 months	**Yes.** MVA adjusted for metastatic status, tumor size, tumor differentiation, and chemotherapy response.
**LRRC15**	Cui 2021 UCLA cohort (69)	NR | Localized + metastatic 31.5(6-77) | NR	NR	IHC	High LRRC15 protein expression: IHC score >2+ IHC score	High LRRC15 expression associated with significantly worse OS: 5-yr OS 72 vs. 45% HR 2.6 (1.4–4.3), p=0.002	**Yes.** MVA adjusted for metastatic status and histologic response.

TABLE LEGEND

Abbreviations: AECM = Albert Einstein College of Medicine; alk phos = alkaline phosphatase; amp = amplification; API-AI = doxorubicin, cisplatin, ifosfamide; BCD = bleomycin, cyclophosphamide, dactinomycin; CCG = Clinical Cooperation Group; CGR = chromosomal genome rearrangement; CN = copy number; COG = Children's Oncology Group; ctDNA = circulating tumor DNA; DFCI = Dana-Farber Cancer Institute; DFS = disease-free survival; diagCPA = ctDNA quantification at diagnosis; EFS = event-free survival; ELISA = enzyme-linked immunosorbent assay; exp = expression; FISH = fluorescence in situ hybridization; G1/G2 = gene expression signature subgroups; GAIN = Genomic Assessment Informs Novel therapy iCat2 study; HR = hazard ratio; ICGC = International Cancer Genome Consortium; IE = ifosfamide + etoposide; IHC = immunohistochemistry; LOH = loss of heterozygosity; MAP = methotrexate, doxorubicin (Adriamycin), cisplatin; MSKCC = Memorial Sloan Kettering Cancer Center; MGH = Massachusetts General Hospital; MTX = methotrexate; MVA = multivariate analysis; NGS = next-generation sequencing; NR = not reported; NS = not significant; OS = overall survival; PCAWG = Pan-Cancer Analysis of Whole Genomes; PFS = progression-free survival; RT-PCR = reverse transcription PCR; RNA-seq = RNA sequencing; SNP = single nucleotide polymorphism; SNV = single nucleotide variant; SV = structural variant; T10 Regimen: High-dose methotrexate; doxorubicin; bleomycin, cyclophosphamide, dactinomycin; selected patients also received cisplatin; TME = tumor microenvironment; ULP-WGS = ultra-low pass whole genome sequencing; VEGFA = vascular endothelial growth factor A; WES = whole exome sequencing; WGD = whole genome doubling; WGS = whole genome sequencing.

Classification of Evidence for Independent Prognostic Value: **Yes =** Multivariable analysis (MVA) adjusted for metastatic status ± other clinical covariates; classifier retained significant association after adjustment. **Partial =** Evidence suggesting prognostic relevance beyond standard clinical factors, but insufficient to establish definitive independence**:** (1) study restricted to specific population (e.g., localized only); (2) stratified analysis by metastatic status without MVA or stratified p-values reported; (3) MVA without adjusting for metastatic status; **No =** Stratified analysis by metastatic status or other sensitivity analyses to assess independent prognostic value or account for potential confounding.

Notes and special notations: The cohorts and number of samples in each reflect only those included in survival analyses. In studies with multiple cohorts, **Bold** text indicates discovery cohort, *italicized* text indicates validation cohort. Frequency refers to the proportion of cases in the high-risk group for a given study. * Data presented from an abstract, with final results pending. [#]70 unique samples, 35 overlapping with Marinoff et. al, 2023 cohort. [^]Includes samples from TARGET(n=59), ICGC(n=5), and TCGA (n=4). [†]Hold-out set not overlapping with OS2006 discovery cohort
